# Detection of Multiple Parallel Transmission Outbreak of *Streptococcus* *suis* Human Infection by Use of Genome Epidemiology, China, 2005

**DOI:** 10.3201/eid2302.160297

**Published:** 2017-02

**Authors:** Pengcheng Du, Han Zheng, Jieping Zhou, Ruiting Lan, Changyun Ye, Huaiqi Jing, Dong Jin, Zhigang Cui, Xuemei Bai, Jianming Liang, Jiantao Liu, Lei Xu, Wen Zhang, Chen Chen, Jianguo Xu

**Affiliations:** State Key Laboratory for Infectious Disease Prevention and Control, Beijing, China (P. Du, H. Zheng, H. Jing, D. Jin, Z. Cui, X. Bai, L. Xu, W. Zhang, C. Chen, J. Xu);; Academy of Sciences of China Institute of Remote Sensing and Digital Earth, Beijing (J. Zhou, J. Liang, J. Liu);; University of New South Wales School of Biotechnology and Biomolecular Sciences, Sydney, Australia (R. Lan);; Collaborative Innovation Center for Diagnosis and Treatment of Infectious Diseases, Beijing (C. Ye, J. Xu);; National Institute for Communicable Disease Control and Prevention, Beijing (D. Jin)

**Keywords:** *Streptococcus* *suis*, streptococci, outbreak, 2005, China, genome epidemiology, piglet breeding company, PBC, bacteria, bacterial infection, industrialized piglet supply system, parallel transmission, zoonoses

## Abstract

*Streptococcus* *suis* sequence type 7 emerged and caused 2 of the largest human infection outbreaks in China in 1998 and 2005. To determine the major risk factors and source of the infections, we analyzed whole genomes of 95 outbreak-associated isolates, identified 160 single nucleotide polymorphisms, and classified them into 6 clades. Molecular clock analysis revealed that clade 1 (responsible for the 1998 outbreak) emerged in October 1997. Clades 2–6 (responsible for the 2005 outbreak) emerged separately during February 2002–August 2004. A total of 41 lineages of *S.* *suis* emerged by the end of 2004 and rapidly expanded to 68 genome types through single base mutations when the outbreak occurred in June 2005. We identified 32 identical isolates and classified them into 8 groups, which were distributed in a large geographic area with no transmission link. These findings suggest that persons were infected in parallel in respective geographic sites.

Human infections caused by *Streptococcus* *suis* have been recognized as a global public health and economic problem in the swine industry (*1*–*3*). These infections afflict persons in close contact with infected pigs or pork-derived products (*4*,*5*). Although sporadic cases of *S.* *suis* infections in humans had been reported worldwide previously, in the summer of 2005, China recorded the largest and most highly diffused outbreak of *S.* *suis* infection in humans, with 215 cases reported and 39 deaths (*6*,*7*). Although the overall case-fatality rate was 18%, it reached 63% among patients with streptococcal toxic shock–like syndrome (*8*,*9*). The causative pathogen was identified as sequence type (ST) 7, which had evolved from ST1 to become a highly virulent strain with epidemic potential. So far, *S.* *suis* ST7 has only been isolated in China (*7*,*9*). The outbreak cases were widely distributed among persons in 203 villages of 12 cities in Sichuan Province. Outbreak investigations by the Chinese Center for Diseases Control and Prevention identified and confirmed 1 case per village in 194 villages (*6*). The outbreak appeared to be caused by pig-to-human direct transmission (*6*). A policy of strictly prohibiting backyard slaughtering was implemented, which ended the outbreak (*3*,*10*). However, the reasons why the outbreak reached such a large scale remained a mystery. 

We used whole-genome sequencing (WGS) to dissect this outbreak through sequencing of 85 isolates from patients and 7 isolates from diseased pigs associated with those patients (*7*). The isolates were divided into 5 clades, which evolved during a 2-year period (2002–2004). The outbreak was probably caused by infected piglets and was amplified by the industrial scale of piglet supply operations in China. These findings uncovered a unique public health threat in China brought about by economic development.

## Materials and Methods

### Isolates

We selected 92 isolates from the 2005 outbreak investigation for genome sequencing and analysis, including 85 isolates from patients and 7 from 6 diseased pigs (*7*), in addition to 2 isolates from a patient and a diseased pig from a 1998 outbreak in Jiangsu Province and 1 from a patient with a sporadic case in Jiangsu in 1999 (*11*). These 85 human isolates represented 39.5% of all cases reported and were distributed among 10 of the 12 affected cities, including the 4 cities accounting for 90.7% (195/215) of all cases and 80% (68/85) of all isolates from patients. We obtained these isolates over a 21-day period during the outbreak, which lasted 41 days.

We used the complete genome sequence of isolate SC84, which was sequenced previously (*12*), as reference. We typed all of the isolates were typed as minimum core genome type 1 (*13*), sequence type 7 (*14*), and showed an identical pulsed-field gel electrophoresis (PFGE) pattern with restriction enzyme *Sma*I (*7*). We obtained information on each patient infected by a given isolate from the enhanced surveillance and investigation we conducted in 2005, including demographic and clinical characteristics and information on type of exposure and place of residence. We did so by searching the original records and database from the previous investigation (*6*,*7*).

### WGS and Analysis of Single-Nucleotide Polymorphisms

We extracted genomic DNA by using Wizard Genomic DNA Purification Kit (Promega, WI, USA). To obtain the genome sequences, we constructed 500-bp libraries and performed WGS by using an Illumina Genome Analyzer IIx system (Illumina, San Diego, CA, USA) to produce 100-bp paired-end reads. We then mapped the high throughput reads to the reference genome of *S.* *suis* strain SC84 (GenBank accession no. NC_012924) by using SOAP2 and detected single-nucleotide polymorphisms (SNPs) by using SOAPsnp version 1.03 (*12*,*15*,*16*). We named the SNPs by using our automatic pipeline described previously (*13*). We constructed the outgroup by using the consensus base of 6 non-ST7 isolates, RC1, YS14, 14636, YS12, S15, and GZ1 (*13*). We conducted recombination analysis by using RDP3 (*17*) and constructed phylogenetic trees by using the Bayesian evolutionary method. We determined the time of divergence of a branch and substitution rates by using BEAST version 1.8.2 (*18*). We found the best-fit evolutionary model for the dataset to be the TN93 model, with a normal distribution of among-site rate heterogeneity and a proportion of invariant sites. We selected a relaxed (uncorrelated exponential) molecular clock and an extended Bayesian Skyline tree prior for the analysis. We performed 3 independent runs with sampling every 10,000 generations of 100,000,000 Markov chain Monte Carlo chains and analyzed the output by using the Tracer module (*18*). We then deposited the sequencing data in the GenBank database (accession no. SRP064815).

### Geographic Information Analysis 

We obtained geographic information for administrative divisions, including the 4 levels of village, town, county, and city, and national and provincial roads and highways in Sichuan. We determined the locations of piglet breeding companies (PBC) in Sichuan in operation before 2005 by using a database maintained by the Animal Husbandry Agency of Sichuan (http://www.scxmsp.gov.cn). We defined geographic distance as the shortest distance between any 2 geographic sites, such as villages, PBCs, or highways. We calculated the means of geographic distances to estimate the ranges which could be affected. We exhibited the geographic distributions of patients, highways, and PBCs by using a visualized digital earth system (VGE-Globa3D) (*19*), which was developed by our geographic information team from the Institute of Remote Sensing and Digital Earth at the Academy of Sciences of China. We performed statistical analyses by using SPSS 16.0 (SPSS Inc., Chicago, IL, USA). We tested pairwise comparison of mean distances by using the Student *t* test and multiple comparisons by using the Kruscal-Walls test. We also tested the association between the geographic distribution of clades from the 2005 outbreak and PBCs in the affected area by using a χ^2^ test. We considered a p value <0.05 to be statistically significant.

## Results

### Genome Sequencing of *S.* *suis* ST7 Outbreak Isolates

We sequenced 85 human isolates and 7 pig isolates from the 2005 outbreak, 2 ST7 isolates from the 1998 outbreak, and 1 isolate from 1999 from a patient with sporadic infection in Jiangsu, where *S.* *suis* ST7 was first isolated in China. We obtained ≈596–1,081 Mb reads of high quality per isolate, which covered on average 284–516 (400.4 +115.7) fold of the complete genome of SC84. All of the assembled genomes covered >98.0% of the reference genome, except for isolate SC218, which covered 94.2%. When mapping these genome sequences to that of SC84, we identified 160 SNPs, with 4–29 SNPs per genome (Technical Appendix 1 Table 1). Overall, we identified 4.1 SNPs per genome, with 1.3 SNPs among 3 isolates from 1998 Jiangsu outbreak and 3.8 SNPs per genome among the 92 isolates from the 2005 Sichuan outbreak. Among the 160 SNPs, 126 were located in 115 genes, including 35 synonymous SNPs and 91 nonsynonymous SNPs, whereas the remaining 34 SNPs were located in intergenic regions. Most genes had 1 SNP only. However, we identified 3 SNPs each for genes SSUSC84_0178 and SSUSC84_1795, which encode a hypothetical protein and serine protease, respectively. The 91 nonsynonymous sites were distributed among 84 genes. The number of nonsynonymous sites exceeded synonymous sites for all Sichuan isolates (6–18 vs. 3–7), leading to high ratios nonsynonymous to synonymous substitutions (dN/dS) ranging from 1.14 to 3.60 and indicating that positive evolutionary pressure during the evolution of ST7 in Sichuan.

### Phylogenetic Relationship of Outbreak-Associated Isolates

**Figure 1:**
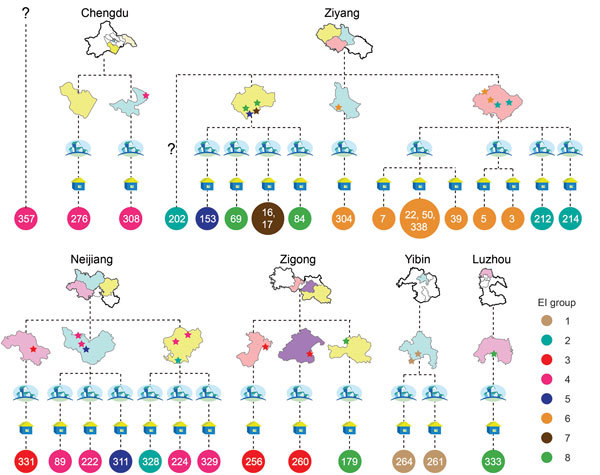
Geographic locations of resident villages of persons infected during outbreak of *Streptococcus suis* identified by whole-genome sequencing as minimum core genome type 1 sequence type 7, Sichuan Province, China, 2005. The 4 levels of administrative divisions (city, county, town, and village) are displayed to show the geographic locations and relationships of isolates. Drawings of the cities and counties are simplified maps of those territories. Towns are depicted with 4 houses, and villages are depicted with 1 house. Stars indicate the locations of patients infected by isolates of the 8 epidemiologically interesting groups, and isolates in the same group are shown in the same color. Circles with numbers represent the isolates, and colors are consistent with the color of the stars. Locations of some isolates were too close to distinguish them on the map (e.g., SC7, SC22, SC50, SC338, and SC39). EI, epidemiologically interesting.

We determined the phylogenetic relationships of the 95 isolates by using Bayesian evolutionary analysis (Technical Appendix 2 Figure 1). We classified these isolates into 6 clades (having 3, 6, 24, 3, 38, and 21 isolates, respectively), which were supported with multiple SNPs. We defined 21 SNPs supporting the clades as clade definition (CD) SNPs (Technical Appendix 1 Table 1; Technical Appendix 2 Figure 1) because they were present in all isolates of a given clade. However, we noted 2 exceptions; CD SNPs A656244G (SSUSC84_0604, synonymous) and A961560C (intergenic) had reverted to the reference base in isolates SC130 and SC218, respectively. We confirmed these 2 nucleotide mutations by PCR and sequencing. Of the 139 non–CD SNPs, 100 SNPs were isolate-specific SNPs, and the remaining 39 SNPs were shared by >2 isolates.

### Emergence of the 5 Clades of *S.* *suis* ST7 Responsible for the 2005 Outbreak 

By using all 160 SNPs and the known isolation dates for the sequenced isolates, we constructed a Bayesian tree to visualize the overall relationships between root-to-tip branch length and the divergence time of the major nodes. We estimated the substitution rate to be 8.58 × 10^−7^ substitutions per site per year (95% highest posterior density 1.98 × 10^−7^ to 1.55 × 10^−6^), corresponding to the accumulation of ≈1.8 SNPs per genome per year. The estimated time to the most recent common ancestor for ST7 lineage in China was May 1996. The 6 ST7 clades appear to have merged successively: clade 1 emerged in October 1997, clade 2 in February 2002, clade 3 in September 2002, clade 4 in November 2002, clade 5 in July 2003, and clade 6 in August 2004. All Sichuan ST7 isolates were of a single origin, and by the end of 2004, ST7 had diversified into 41 lineages and saw a rapid expansion to 68 genome types through single base mutations by June 2005, when the outbreak occurred (Technical Appendix 2 Figure 1). Therefore, substantial diversity had already developed within ST7 before the outbreak.

### Geographic Distribution of Outbreak-Associated Isolates

We classified 32 of 92 outbreak-associated isolates (25 from 25 patients and 7 from 6 diseased pigs) into 8 genome types, which we termed as epidemiologically informative (EI) groups (Figure 1). The whole genome sequences of all isolates in a given EI group were identical (i.e., whole-genome identical isolates), which provide critical information for epidemiologic tracing. The EI groups contained 2–8 isolates. All except 2 human isolates from these EI groups had geographic information associated with them based on the patients’ (or diseased pigs’) place of residence. All EI groups except EI 1 and EI 7 were distributed across different counties or cities. Most EI 6 isolates were confined to 1 county but spread across 5 villages in 2 different towns. In total, these isolates were distributed in 26 villages, 24 towns, 13 counties, and 6 cities. No epidemiologic evidence indicated that these whole-genome identical isolates from different towns were a result of direct transmission. The EI groups were highly unlikely to have originated from a single infectious source for the outbreak. To account for such strain distribution patterns, the most likely scenario was that these EI groups had been distributed widely before the outbreak (Figure 1). To achieve such a wide distribution, the most likely explanation is that piglets were infected with these strains in the breeding companies before distribution to the backyard farmers.

**Figure 2:**
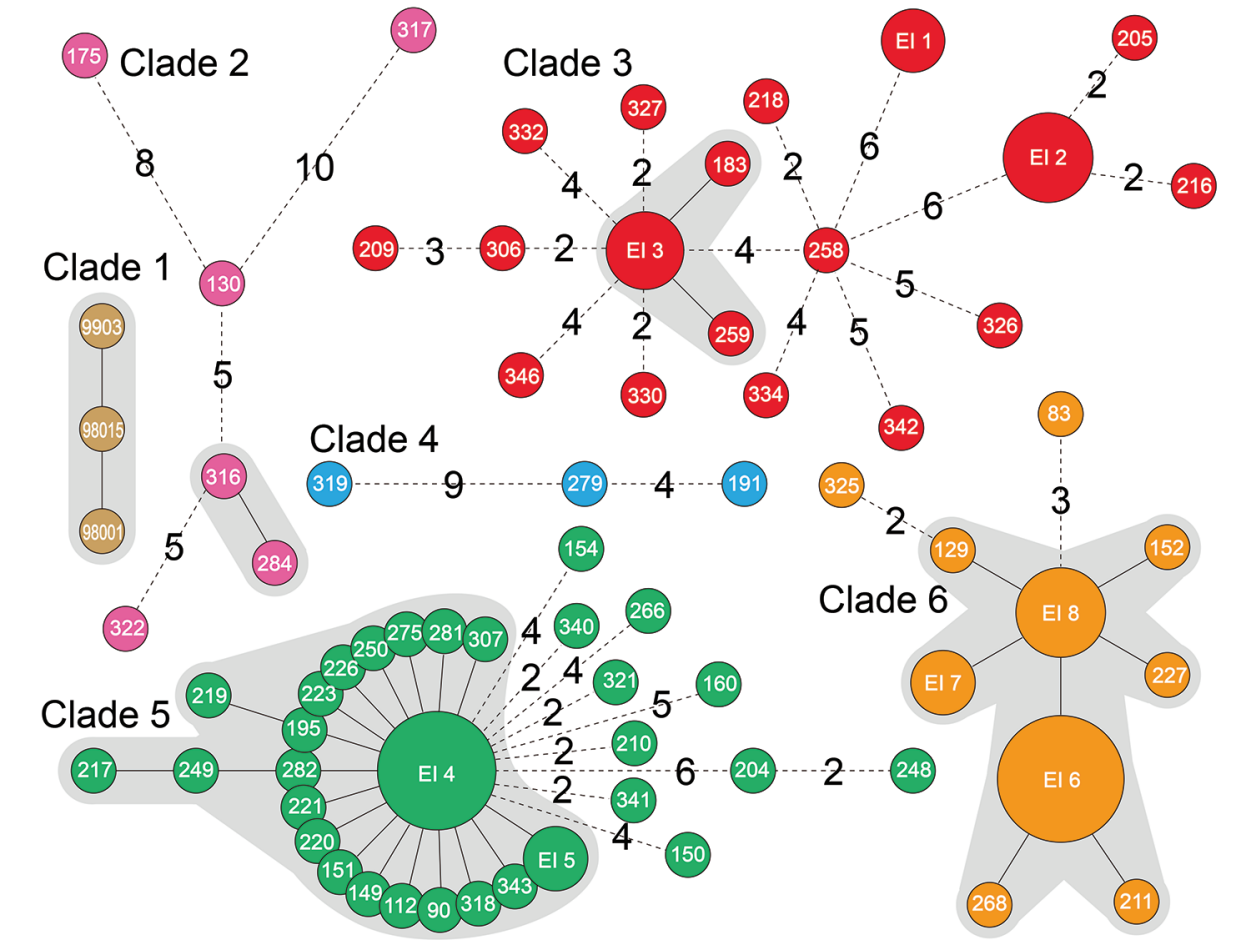
Minimum spanning tree of 6 clades of *Streptococcus* *suis* minimum core genome type 1 sequence type 7 implicated in outbreak of human infections, Sichuan Province, China, 2005. Solid black lines indicate only 1 single-nucleotide polymorphism between the 2 isolates. Differences of >1 SNP between isolates are displayed by dashed black lines with numbers. Numbers in circles are names of isolates. Epidemiologically interesting (EI) group 1: SC261 and SC264; EI 2: SC202, SC212, SC214, and SC328; EI 3: SC256, SC260, and SC331; EI 4: SC89, SC222, SC224, SC276, SC308, SC329, and SC357; EI 5: SC153 and SC311; EI 6: SC3, SC5, SC7, SC22, SC39, SC50, SC304, and SC338; EI 7: SC16 and SC17; EI 8: SC69, SC84, SC179, and SC333.

We further analyzed the geographic distribution of isolates with a genome difference of 1 SNP (Figure 2). This spread scenario might also apply because the single SNP diversity might have developed during the raising of the infected piglets by backyard farmers. We identified 2, 11, 28, and 19 isolates from clades 2, 3, 5, and 6, respectively, that can be grouped into 4 clonal complexes with 1 SNP difference (in addition to the EI groups), including almost two thirds (60/92) of outbreak-associated isolates (Figure 2). We further examined geographic distribution of the members of the clonal complexes. Except for 7 isolates that had no information associated with them regarding village, town, county, or city level, the clonal complexes were distributed among 49 villages, 42 towns, 22 counties, and 8 cities. This finding of the same clonal complexes (i.e., high genetic relatedness) with a wide geographic distribution lends further support to our hypothesis that the piglets in these cases had been distributed widely before the outbreak occurred. This scenario is likely given that the piglets were probably colonized by the pathogen before sale or distribution.

**Figure 3:**
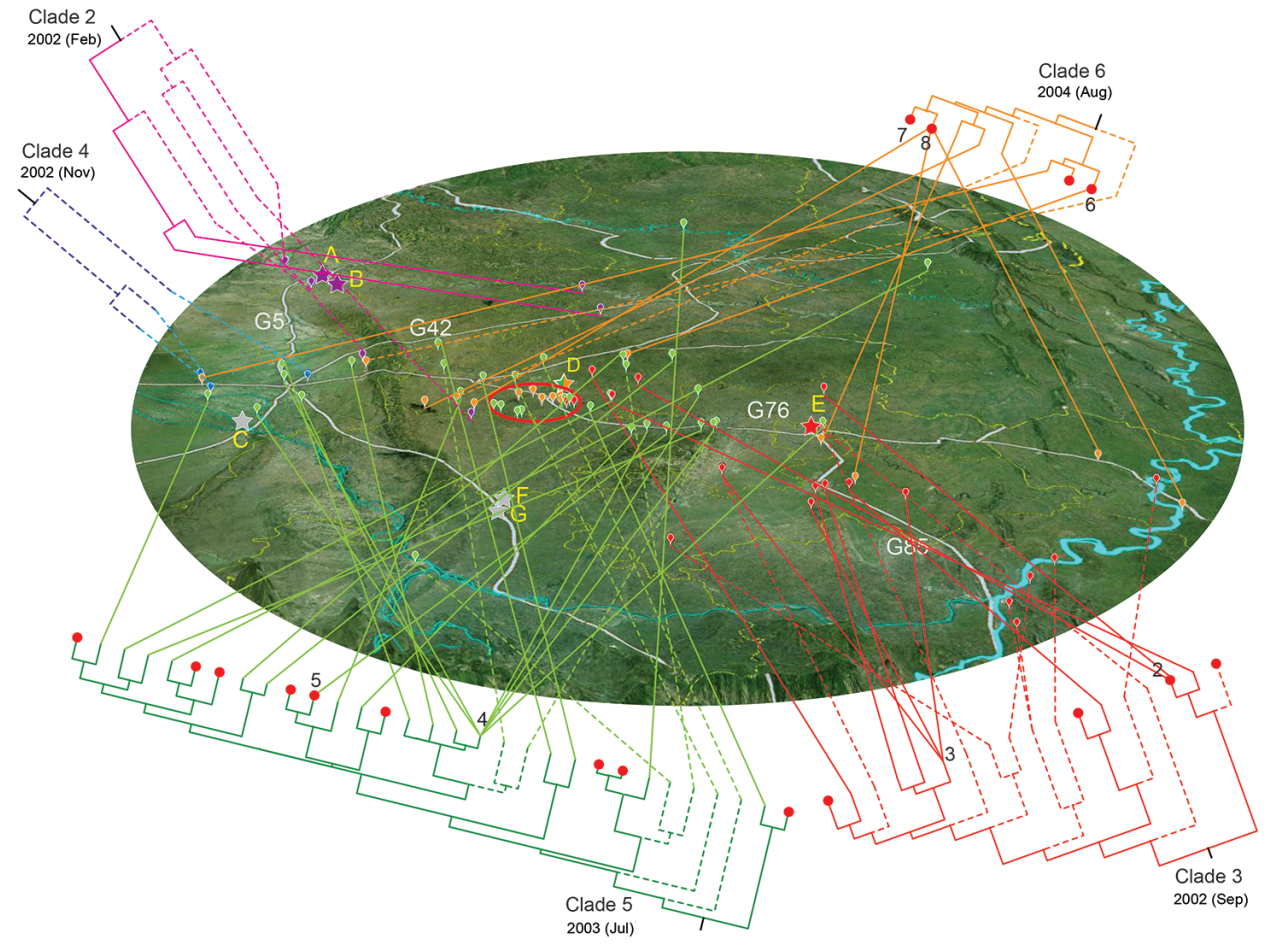
Geographic spread of the 5 outbreak clades of *Streptococcus* *suis* minimum core genome type 1 sequence type 7 before and during outbreak of *S. suis* human infections, Sichuan Province, China, 2005. Phylogenetic relationship of isolates within a clade is detailed in Technical Appendix 2 Figure 1). Lineages that developed before 2005 are marked with dashed lines. Clades are displayed separately around the map for better visualization of their geographic distribution. Locations are marked by bubbles in different colors (clades 2–6 are purple, red, blue, green, and orange respectively). The isolates in the same epidemiologically interesting group are collapsed at the tip of the tree and identified by their group number. Bubbles in the red ellipse on the map represent isolates from the most concentrated outbreak region of Ziyang city; red plots on the tree represent these isolates; stars represent the piglet breeding companies.

Our geographic and phylogenetic analyses support this hypothesis. The molecular clock analysis estimated that all Sichuan ST7 isolates were diversified into 41 lineages by the end of 2004, nearly 6 months before the outbreak, and rapidly expanded into 68 genome types, which were distributed throughout vast geographic areas (Figure 3). Typically, the production period of raising piglets until they are grown pigs fit for slaughter is ≈6 months. Therefore, those ST7 lineages most likely colonized the piglets before they arrived at the backyard farmers (Figure 3).

### Association of Disease Spread with Major Roads and Piglet Breeding Companies

**Figure 4:**
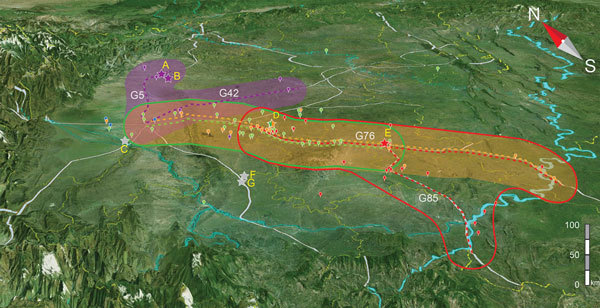
Geographic locations and affected region of major highways and piglet breeding companies associated with outbreaks of human infection with *Streptococcus* *suis* minimum core genome type 1 sequence type 7, Sichuan Province, China, 2005. Colored bubbles represent Sichuan clades 2–6 (purple, red, blue, green, and orange, respectively). Stars represent the piglet breeding companies, dashed lines represent the associated highways, and colors are consistent with the clades they are related to. Purple shadow, area in red line, area in green line, and orange shadow represent the affected regions related to clades 2, 3, 5, and 6, respectively.

When we plotted the locations of villages in which 72 of the patients and six of the deceased pigs had resided (village information for 13 patients was missing), we saw clearly that most of the villages were along the major roads or highways (Figure 4). Therefore, we hypothesized that the pathogen was carried by piglets that were traded from the PBCs to backyard farmers by using public ground transportation (*20*). To test this hypothesis, we analyzed the geographic distances between the patients’ resident villages and major PBCs and between the patients’ resident villages and highways that existed at that time. Seven major PBCs (A–G) were in operation around the outbreak periods; of these, PBCs A and B and PBCs F and G were close to each other (within a range of 10 km), so we treated each pair as a single entity (i.e., PBC A/B and PBC F/G) (Technical Appendix 1 Table 2).

Because piglets typically were distributed locally, we used the shortest distance of the patients’ resident villages to the nearest PBCs and highways to determine the likely sources of the clades (Technical Appendix 1 Table 2). Notably, clade 4 had only 3 isolates for computation of the association. When the mean distance of each clade to nearest highways were compared with that of all isolates to each other, the association of clades 2, 3, 5, and 6 with their nearest respective highway (G5 and G42, G76, G76, and G76) were statistically significant (Technical Appendix 1 Table 3). Most cases were in patients who lived within 50 km of the nearest highway (Figure 4; Technical Appendix 2 Figure 2). Therefore, transportation of the piglets through the highways probably played a role in the spreading of the disease. We used similar methods to test the association of clades with PBCs. Clades 2, 3, 5, and 6 were statistically significantly associated with PBC A/B, PBC E, PBC D, and PBC D, respectively (Technical Appendix 1 Table 3; Technical Appendix 2 Figure 2).

## Discussion

In this study, genome epidemiology was used to obtain a high-resolution dissection of the largest and most highly diffused human infection outbreak of *S.* *suis*, which occurred in Sichuan, Province, China, in 2005. Phylogenetic analysis with whole genome sequences divided the outbreak isolates into 68 lineages and 5 clades, showing substantial diversity among the outbreak isolates. The outbreak was most likely caused by the distribution of infected piglets from industrialized PBCs to farmers’ backyards across wide geographic regions. The wide distribution of piglets in the region and the massive backyard slaughtering of diseased pigs in a short period led to numerous parallel transmissions from infected pigs to humans (*21*). The outbreak in swine peaked around July 20, 2005, and *S.* *suis* caused 98% of the deaths among these pigs (*6*).

Phylogenetic analysis of the 92 isolates from the Sichuan outbreak, including 85 human isolates, showed that these isolates can be divided into 5 clades. The clades diverged at various points in time during February 2002–August 2004. Thus, the diversity was developed years before the outbreak and not during or months before the outbreak. However, most (59) of the outbreak isolates belonged to clade 5 and were closely related to clade 6 isolates.  In addition, 8 groups of isolates (consisting of a total of 32 isolates) were identical in genome sequences; these were divided into 8 EI groups because they allowed epidemiological tracing given that identical isolates from diverse geographic regions implied the same source of infection, which most likely was the PBCs.

The timing of the outbreak was consistent with a scenario in which piglets were infected at the source rather than in the backyard. Piglets take 6 months to grow to adult pigs for slaughtering. The incubation period for the outbreak was nearly 6 months. The observation of isolates with 1 SNP difference (Figure 2) also supports a common source of *S.* *suis* infection given that our estimated mutation rate is 1.8 SNPs per genome per year. *S.* *suis* can also cause disease in pigs. The human outbreak followed a large swine outbreak that killed ≈10,000 backyard pigs, further indicating that the outbreak was caused by infection in pigs (*6*). The identification of 41 lineages belonging to 5 clades suggests that the diversity of the *S.* *suis* strain was developed in the PBCs, thus explaining the heterogeneity of strains from the same source before the outbreak.

Our mapping of the transportation route with the locations of the 5 *S.* *suis* clades and PBCs further support the distribution of infected piglets. In particular, PBC D was associated with clades 5 and clade 6. However, no isolates were available from the company to confirm the link. Overall, our integration of the genomic data and the geographic data explains the highly diffused pattern of the Sichuan outbreak.

Ye et al. (*7*) found that the small *S. suis* outbreak in Jiangsu in 1998 reported by Zhu et al. (*11*) was caused by ST7; therefore, they have suggested that the ST7 strains involved in the Sichuan outbreak originated phylogenetically from Jiangsu and were spread through interprovincial import of breeder pigs to Sichuan (*11*). The inclusion of 2 isolates associated with Jiangsu outbreak confirmed the evolutionary link of the 2 outbreaks. The Jiangsu outbreak isolates were clustered together as clade 1 and diverged earliest. The origin of the Jiangsu and Sichuan strains dates back to 1996, and ST7 very likely spread across the provinces through carriage by breeder pigs.

Previous studies found that all of the outbreak isolates were highly homogenous and belonged to ST7 (*7*). Further analysis with PFGE, which is considered to be a gold standard, showed that the 2005 outbreak isolates belonged to the same PFGE pattern (*7*). During the outbreak investigation in 2005, the outbreak was found to be caused by a single homogenous clonal strain (*7*). Our genomic data provided much higher resolution to reveal a high level of heterogeneity within the outbreak involving 5 clades that had developed well before the outbreak occurred. Our findings also exposed the inadequacy of PFGE in tracing *S.* *suis* ST7 transmission.

The outbreak was an unforeseen consequence of economic development. To increase pork production, imported pig breeds replaced local breeds in China, and piglets were produced through large companies and distributed to backyard farmers. This combination of practices has been commonplace in Sichuan Province and many other parts of China. The farmers receive piglets from large industrial-scale companies to raise in their small backyards under poor hygienic conditions. In Sichuan, a sizable swine population was found in small backyard farms, and nearly every family was keeping a few swine at the time of the outbreak. The combination of a highly industrialized piglet supply system and the farmer’s backyard animal raising practices might have created a high risk for infectious disease outbreaks of unprecedented scale in terms of the number of persons infected and the geographic spread, posing an even greater public health threat (*22**,**23*). A pathogen-free supply of piglets and improved hygiene for backyard farmers could help prevent such outbreaks (*23**–**26*). Alternatively, disease monitoring at the PBC level would be a very effective outbreak-prevention strategy. Clades 5 and 6 were associated with PBC D and accounted for the majority of the outbreak isolates. Therefore, PBC D was likely to be the primary contributor to the outbreak. Clades 5 and 6 shared the most recent common ancestor (Figure 1) with the time of divergence of the clades dating back to 2003, so piglets at PBC D probably were infected by *S.* *suis* ST7 for >2 years before the outbreak. Because ST7 also causes disease in pigs, monitoring and intervention at the PBC level could have averted the outbreak, underscoring the importance of disease monitoring at its source for zoonotic human infections.

In conclusion, the Sichuan outbreak of *S.* *suis* in humans was caused by the parallel transmission of infection from pigs to humans through distributed pig farming. The combination of centralized industrial-scale supply of infected piglets by PBCs and the backyard animal raising practices of farmers has created a unique environment for the incubation of a large outbreak. A pathogen that formerly only caused sporadic disease has now evolved to become a major threat to human health. Our findings provide important insights into *S.* *suis* epidemiology and demonstrate that novel intervention strategies are required for the prevention of such outbreaks.

Technical Appendix 1 Clades of *Streptococcus suis* minimum core genome type 1 sequence type 7 and epidemiologically interesting group–specific single nucleotide polymorphisms, geographic distance of human isolates of *S. suis* clades to all piglet breeding companies and highways, and mean geographic distance of each clade of *S. suis* to the nearest piglet breeding company and highway, Sichuan Province, China, 2005.

Technical Appendix 2 Phylogenetic relationship and evolution timescale of Streptococcus suis minimum core genome type 1 sequence type 7 and percentage distribution of S. suis human infection cases by distance to the nearest piglet breeding company and highway, Sichuan Province, China, 2005.
