# Supplementary material for: Detection of Multiple Parallel Transmission Outbreak of Streptococcus suis Human Infection by Use of Genome Epidemiology, China, 2005
Source: Emerg Infect Dis. 2017 Feb;23(2):204–11. doi: 10.3201/eid2302.160297 (PMC5324821; doi:10.3201/eid2302.160297)
Supplement: Technical Appendix 2 — Phylogenetic relationship and evolution timescale of Streptococcus suis minimum core genome type 1 sequence type 7 and percentage distribution of S. suis human infection cases by distance to the nearest piglet breeding company and highway, Sichuan Province, China, 2005. [file 16-0297-Techapp-s2.pdf]

Article DOI: http://dx.doi.org/10.3201/eid2302.160297

Detection of Multiple Parallel Transmission Outbreak of *Streptococcus suis* Human Infection by Use of Genome Epidemiology, China, 2005

Technical Appendix 2

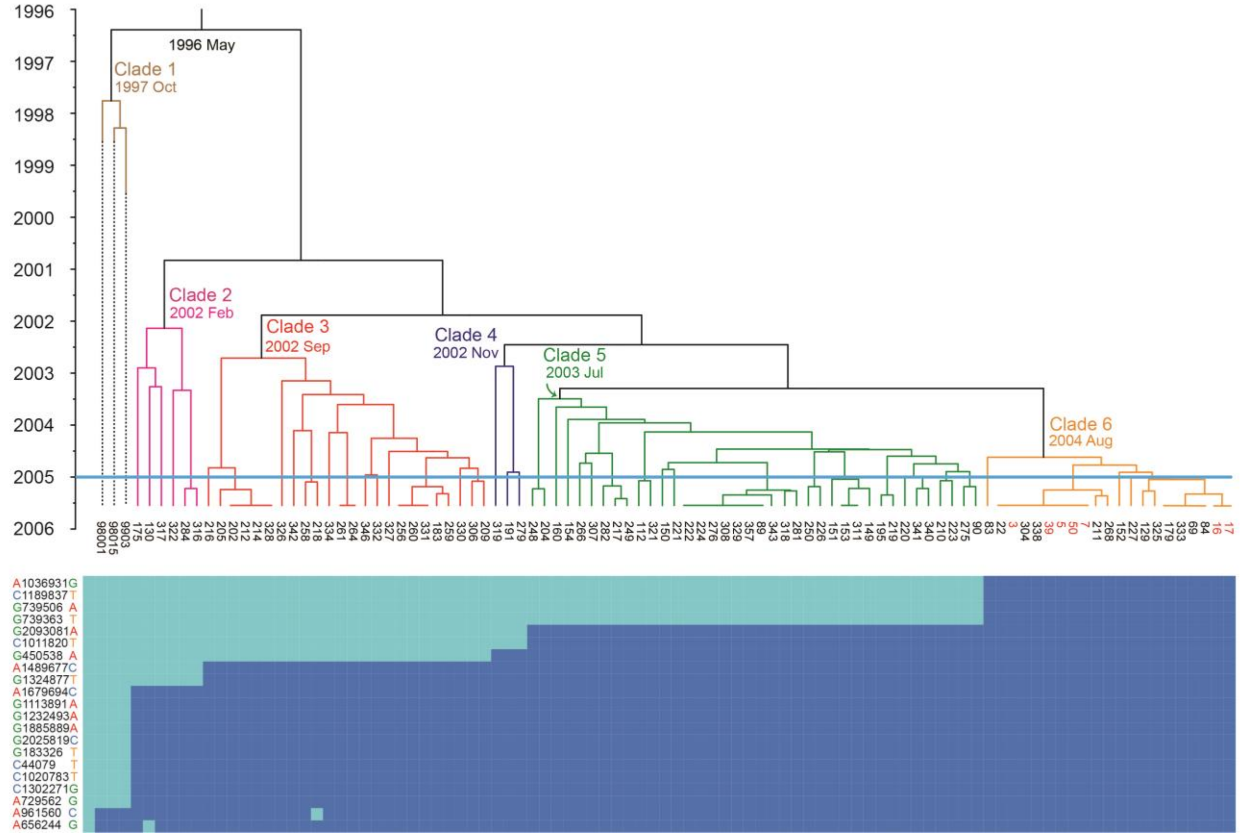

Technical Appendix 2 Figure 1. Phylogenetic relationship and evolution timescale of *Streptococcus suis* minimum core genome type 1 sequence type 7, Sichuan Province, China, 2005. Bayes tree of the isolates is at the top of figure. The 6 clades (clades 1–6) are showed in brown, purple, red, blue, green, and orange, respectively. The inferred emerging time of each clade is indicated on the branches by year

and month. The distribution of 21 clade definition single nucleotide polymorphisms are shown in the heat map at the bottom of the figure. The mutated nucleotides and sites are shown at left, which is displayed with the blocks in deep blue. The names of isolates from diseased pigs are in red.

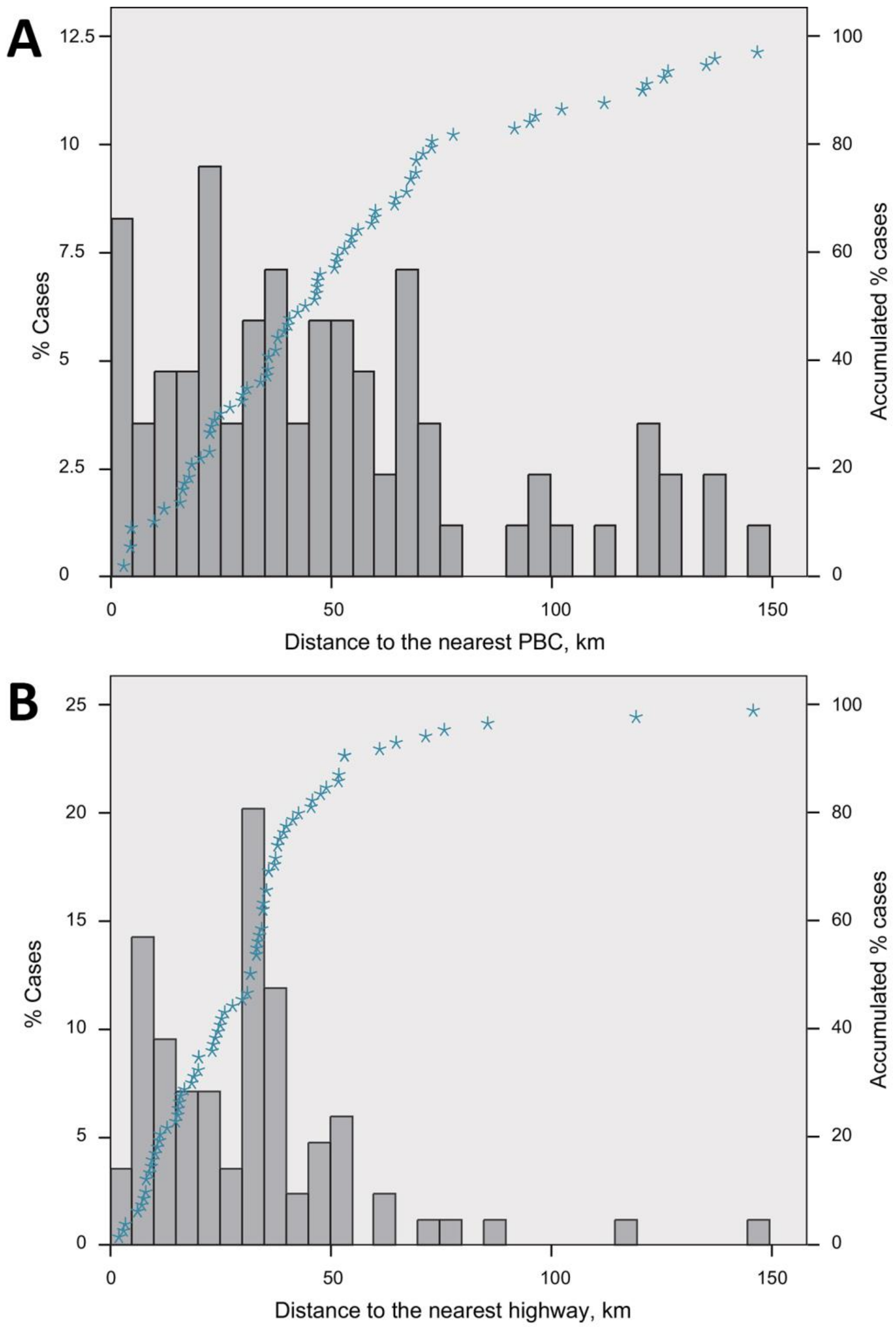

Technical Appendix 2 Figure 2. Percentage distribution of cases of human infection with *Streptococcus suis* minimum core genome type 1 sequence type 7 by distance to the nearest piglet breeding company (A) and highway (B), Sichuan Province, China, 2005. The x axes is the geographic distance of the

patient’s resident village to the nearest piglet breeding company or highway. The gray bars represent the percentage of cases (left y axes), and the asterisks represent the accumulated percentage of cases (right y axes). PBC, piglet breeding company.
